# Letter from Dr. Vernois

**Published:** 1839-07-27

**Authors:** 


### FOREIGN CORRESPONDENCE.

### LETTER FROM DR. VERNOIS. No. II.

Paris, *May 20th*, 1839.

To the Editors of the Medical Examiner.

After an atmosphere of violent and protracted storms, the medical horizon has cleared up, and resumed its ordinary color. The Academy, where tumult, and passion, and, at times, eloquence so lately prevailed, in the midst of the discussion on the motor and sensitive nerves, is now observing its classic silence; and, unfortunately, from the shock of so many conflicting opinions, light has not broken forth. The Academy of Medicine, as it is at present constituted, is, perhaps, the worst constituted body to advance the interests of science. There every one, enlightened or not upon the nature of the question, feels called upon to utter his opinion, and give the results of his long experience; and the discussions, thus interfered with, degenerate into idle declamations, if, indeed, they do not end in personal disputations, disgraceful to the combatants, and unworthy the gravity of an Academy. A fruitful field for such discussions has offered itself for some time past—the subject of poisonous and arsenious acid, the discussion on the functions of the nerves, the election of a member in the section of anatomy and physiology, (which resulted in the choice of an orthopedist,) all these were admissible topics for the eloquence of the illustrious members. The Academy is, for the time, in a state of repose. We must, therefore, elsewhere seek a subject of our letter. We should have wished to speak of the *concours* of the Faculty of Medicine, but the echo of its amphitheatres has ceased to be heard. The competitors, in the retirement of the closet, devoting all their energies to the working out of that which chance has allotted them. In this allotment, as in all matters the subject of hazard, both good and bad subjects are to be found. By the side of questions involving enlarged novel views, and full of interest, are to be found topics dry, empty, and nearly barren of interest. We shall devote a special letter to the study of this clinical trial, which will probably be rendered not a little curious and brilliant.

But, beyond the limits of the Academy and School, are to be found other (we were going to say, the great body of) zealous and industrious workers, full of devotion to science and the progress of medicine. The hospitals shall afford us an example. M. Piorry, well known for his labours in semeiology, and his lately published system of the classification and denomination of diseases, a few days since opened his clinique at the hospital of La Pitié. This year he proposes, in his lectures, to follow the subject of diseases of the blood. It is useless to remind all who have kept pace with the progress of the science, the difficulties of this topic, and the dearth of clinical labours upon the subject. There are, therefore, evinced both devotion and medical tact in the pursuit of such investigations. Undoubtedly chemistry, physics, and the microscope, have shed some light upon the subject: we are all familiar with the researches of M. Lecann, of M. Herraut, (d'Alfort, of MM. Bouillaud, Donne, Mault, &c. But there is a wide distance between the knowledge of facts, developed by these inquiries, and such as are demanded by practical medicine. How often does theory fail us at the bedside of the patient! And how few are the observations which point out clearly the connection between an alteration in the constitution of the blood, and the well-marked symptoms of any disease? It is to the establishment of this important relation, by ascertaining the elements of its composition, from the observation of the minutest clinical facts, that M. Piorry means to devote his summer tour of duty. As an appropriate introduction to the future details on this subject, which we may gather from the instruction of M. Piorry, I propose to offer you a succinct analysis of a work on the same subject, of no great celebrity, though well entitled to it, both from the novel manner in which the lesions of the blood are treated of, and the equally novel therapeutic precepts deduced therefrom. I allude to the work of M. Denis de Commercy, entitled, "An Essay on the Application of Organic Chemistry, to the Physiological Study of the Blood of Man, and to the Physiologico-Pathological, Hygienic, and Therapeutic Study of the Diseases of this Fluid."

It is by the application of the chemical results obtained in the laboratory that M. Denis attempts to illustrate the mechanism of the vital organization.

He recognises but a single order of phenomena in the universe, and this order is governed by general laws which are common to all bodies, whether inert or animated. The latter, without doubt, owe their life to a principle, which may be called vital, but whose sole action is that of exciting, of directing, in the body animated by it, the physical and chemical powers inherent in matter, without deranging or changing the nature of their effects.

As regards the special laws of this principle, they belong to the domain of psychology and metaphysics; so that positive physiology, according to M. Denis, rests exclusively upon the data furnished by physics and chemistry.

Since the extinction of vitality does not produce instant modification in organised matter,—for the dead body may be looked upon as a machine merely in repose, which a moment before was in the full exercise of its functions and still preserving the combination of wheels and springs so lately active, and is deficient only in the secret cause of their harmonious movement, the ensemble of the products of physical and chemical decomposition, artificially induced, may, according to M. Denis, be regarded as the exact representation of the material state of life; and we may safely admit that death does not rob the tissues and humours of those chemical properties, which, when exercised under the influence of vitality, gave rise to the various organic functions.

Hence it follows that chemical research alone can enable us to unravel the intricacies of organization and instruct us in those subtile reactions which are constantly going on during the existence of the animated being.

Consonantly, then, with these views, physiology, the knowledge of the physical structure and chemical composition of organs and humours, as well as the physical and chemical effects apparent in each individual, must be deferred so long as these effects are brought about through the influence of vitality.

Pathology and Therapeutics, therefore, would have for the objects of their study, the former, instruments and various physico-chemical reaction, the latter, the rectification of these instruments through physico-chemical means.

After setting forth this doctrine, which we do not pretend to defend in every particular, M. Denis, in the first part of his essays, gives himself up to chemical researches in the human blood. Having this object ever in view, he examines successively the substances which enter into the composition of the blood, and the processes employed for their elimination, as merely for determining in an analysis those which may be considered as immediate constituents. These researches are purely chemical.

Let us observe, that M. Denis demonstrates in them that fibrine and albumen are one and the same thing; that the salts of the blood perform a most important office in this humour and in the organs; that the yellow biliary matter evident in the fluids and tissues of those labouring under jaundice, exists also in healthy blood; that the globules of the blood have an albuminous nucleus, etc.

This part terminates with an exposition of the method of analysing the blood.

The second contains deductions furnished by the preceding researches.

The author first determines what the composition of the blood in a healthy individual should be, and establishes the theory of molecular phenomena which take place in it during the exercise of the function.

To this end he gives the analysis of the globules and serum. He finds that the varieties of healthy blood result from the mixture of these in different proportions; that the globules and serum have a respectively identical composition.

He follows them into the body, and infers, from reactions studied in the laboratory, those which their constituent substances should have in the living economy.

Thus much for physiology.

In continuation, M. Denis enters upon a similar investigation into unhealthy blood. After a comprehensive glance at the affections of this fluid, he gives the composition of the buffy, and of the tarry blood, of the incoagulable, of the thick, the watery, the blood of jaundiced subjects, blood with colourless serum, of white blood, and of the blood of those affected with cholera.

The greater part of the facts which show their composition have been developed by M. Denis, and form a series of discoveries *most interesting for science.*

The examination of the phenomena which seem to result from an alteration of the blood, leads the author to considerations on the formation of the buffy coat which occasionally covers the blood, on croup, typhoid affections, scurvy, plethora, anemia, jaundice, and cholera.

Then follow some very ingenious therapeutic deductions regarding the employment of means calculated to maintain the blood in its normal state, and on the rational treatment which chemical researches should induce us to adopt in diseases depending on an altered state of the fluid.

From this brief analysis may readily be conceived the importance of M. Denis's labours, and of what utility the perusal and study of these investigations will be to those who wish seriously to inquire into the lesions of the blood, and into the means for remedying them.

